# Results of relugolix/estradiol/norethisterone acetate combination therapy in real-world clinical practice: Effectiveness, tolerability, and factors influencing discontinuation

**DOI:** 10.1016/j.eurox.2025.100442

**Published:** 2025-12-30

**Authors:** A. Santalla-Hernández, M. Naveiro-Fuentes, N. Esquinas-Orellana, LM Benítez-Cejas, M. García-Rivera, J. Fernández-Parra

**Affiliations:** Obstetrics and gynaecology department. University Hospital Virgen de las Nieves. Granada. Spain

**Keywords:** Estradiol, Norethisterone acetate, Relugolix, Uterine fibroids, Bleeding, Symptom severity score

## Abstract

**Objective:**

To evaluate the real-world effectiveness, safety, and tolerability of relugolix/estradiol/norethisterone acetate combination therapy (relugolix CT) for managing symptomatic uterine fibroids in routine gynaecological practice.

**Methods:**

A retrospective, observational study was conducted at a tertiary hospital in Spain between June 2023 and January 2025. Adult women with symptomatic uterine fibroids treated with relugolix CT were included, either as a long-term treatment or preoperative management. Clinical outcomes were assessed at baseline, 6 months, and in a subset of patients, at 12 months. Primary endpoints included changes in total bleeding days, heavy menstrual bleeding (HMB) days, and UFS-QoL symptom severity scores (SSS), as well as treatment adherence and adverse events.

**Results:**

Among the 142 women who initiated treatment, Relugolix CT significantly reduced total bleeding days (from 12 to 2.2), HMB days (from 7.1 to 0.4), and UFS-QoL SSS (from 27.9 to 15.1) at 6 months (p < 0.05). Amenorrhea was achieved in 52.7 % at 6 months and 66 % at 12 months. Better clinical outcomes were linked to continued treatment. Adverse events were reported in 34.5 % of patients, primarily abdominal pain and vasomotor symptoms. Bone densitometry at 12 months showed no osteoporosis and mild osteopenia in a few patients. Discontinuation was most commonly due to planned surgery or perceived lack of efficacy.

**Conclusion:**

Relugolix CT demonstrates strong real-world effectiveness and tolerability for managing symptomatic uterine fibroids, with marked improvements in bleeding and quality of life, and a favourable safety profile.

## Highlights

• Relugolix CT reduced bleeding days and UFS-QoL SSS in women with uterine fibroids.
• Amenorrhea achieved in 52.7 % at 6 months, rising to 66 % at 12 months.
• Adverse events were mostly mild; no cases of osteoporosis at 12 months.
• Better outcomes were linked to longer treatment duration with Relugolix CT.

## 1 Introduction

Uterine fibroids are the most common benign uterine tumors affecting women of reproductive age [1]. Although many fibroids are asymptomatic, approximately one-third of cases present with clinically significant symptoms, primarily heavy menstrual bleeding (HMB) and pressure-related discomfort, which can severely impair quality of life [2].

Surgical intervention has traditionally been the cornerstone of fibroid treatment. However, in recent years, there has been growing interest in the development of conservative therapeutic options aimed at avoiding surgery, particularly among women who wish to preserve their uterus or reduce surgical risks [3]. Among these novel therapeutic approaches, oral gonadotropin-releasing hormone (GnRH) receptor antagonists have emerged as a significant innovation. These agents suppress the hypothalamic–pituitary–gonadal axis, inducing a hypoestrogenic state that reduces fibroid size and alleviates associated symptoms [4].

The first GnRH antagonist approved for clinical use in this context is a fixed-dose combination therapy (CT) of relugolix (40 mg), estradiol (1 mg), and norethisterone acetate (0.5 mg) (Relugolix CT, Ryeqo®). Relugolix exerts a direct antagonistic effect on GnRH receptors, while the “add-back” therapy with estradiol and norethisterone mitigates both the short- and long-term adverse effects of estrogen deprivation, particularly vasomotor symptoms and loss of bone mineral density (BMD). In the case of uterine fibroids, it may be indicated in two primary clinical scenarios: as a preoperative therapy to reduce bleeding and fibroid size prior to planned surgical intervention, or as a long-term treatment for symptom control in women nearing menopause for whom surgery is neither imminent nor desired [5]. While its efficacy and safety have been demonstrated in randomized controlled trials, such as the LIBERTY 1 and 2 studies 6, 7, real-world data on its effectiveness, tolerability, and adherence in routine gynecological practice remain lacking. To date, no published studies have evaluated Relugolix CT outcomes in unselected populations under real-world conditions using standard outpatient gynecology follow-up protocols.

The objective of this study was to assess the clinical effectiveness, safety, and tolerability of Relugolix CT in women with symptomatic uterine fibroids treated at a tertiary care center. Specifically, we aimed to analyze its impact on menstrual bleeding and symptom severity, determine rates and reasons for treatment discontinuation, and identify factors associated with adherence in routine outpatient gynecological care.

## 2 Materials and methods

A retrospective, observational study was conducted at the Department of Gynecology at Hospital Universitario Virgen de las Nieves (Granada, Spain) in women with symptomatic uterine fibroids under routine clinical practice. The study period ranged from June 2023 to January 2025 and included adult women diagnosed with uterine fibroids confirmed by transvaginal ultrasound, who presented with symptoms such as heavy menstrual bleeding and/or bulk-related complaints in whom other causes of heavy menstrual bleeding, such as endometrial polyps or adenomyosis, had been ruled out by ultrasound examination. Based on clinical judgment, Relugolix CT was prescribed either as a preoperative treatment for patients who had already been scheduled for surgery (to reduce heavy menstrual bleeding while the patient was awaiting surgery) or as a long-term management option. Patients who presented contraindications to drug administration as specified in the approved prescribing information, or who declined participation in the study, were excluded

For each patient, baseline data were collected, including age, previous hormonal therapy, number of fibroids, FIGO classification, and the maximum diameter of the largest fibroid. Menstrual symptoms were assessed using the total number of bleeding days, number of days with heavy menstrual bleeding (HMB), and the Symptom Severity Score (SSS) from the validated UFS-QoL questionnaire [8]. Additionally, data were collected regarding the timing of the prescription (i.e., whether it occurred during the early or later stages of the drug’s availability) and the therapeutic intent. Specifically, Relugolix CT was prescribed either as an indefinite medical treatment or as a bridging therapy prior to scheduled surgical intervention.

Clinical follow-up visits were conducted at 6 months after treatment initiation and, in a subset of patients, also at 12 months, coinciding with the routine schedule used for treating symptomatic uterine fibroids. At each visit, clinical response was assessed based on changes in bleeding patterns and SSS scores, and the occurrence of amenorrhea, defined as the absence of menstrual bleeding in the preceding three months, was recorded. Adverse events, treatment discontinuation, and reasons for withdrawal were also documented.

The study was approved by the BIOMEDICAL RESEARCH ETHICS COMMITTEE OF THE PROVINCE OF GRANADA under protocol number SICEIA-2025–001823.

Statistical analyses included descriptive statistics to summarize the baseline character-istics and clinical outcomes, with quantitative variables expressed as means and standard deviations (SD) or medians and interquartile ranges, and categorical variables as absolute and relative frequencies. Comparisons of clinical outcomes between baseline and follow-up were conducted using paired Student’s t tests or Wilcoxon signed-rank tests, depending on data distribution. For outcomes assessed at three timepoints (baseline, 6 months, 12 months), repeated-measures Friedman tests were applied. Comparisons between independent groups (e.g. treatment continuation versus discontinuation, preoperative versus indefinite use, early versus later prescription timing) were made using independent t- tests or Mann-Whitney U tests for continuous variables, and chi-square or Fisher’s exact tests for categorical variables. A two-sided p-value < 0.05 was considered statistically significant.

To identify independent predictors of treatment efficacy, two multivariable linear regression models were constructed, with total bleeding days and days of HMB at 6 months as dependent variables. The following covariates were included in both models: treatment continuation (yes/no), patient age, number of fibroids, fibroid type (submucosal, intramural, subserosal), and maximum fibroid diameter. Estimates were reported as coefficients with 95 % confidence intervals (95 %CI) and corresponding p-values. A p-value of < 0.05 was considered statistically significant. All analyses were performed using the R Commander statistical software.

## 3 Results

A total of 142 patients initiated treatment with Relugolix CT. At the 6-month follow-up, 81 patients (57.0 %) still had an active medical prescription for Relugolix CT. At 12 months, 52 patients (43.7 %) continued to have the prescription active. Notably, a substantial proportion of those who no longer had an active prescription at 6 months had completed the treatment as planned in the context of scheduled surgery (17 patients). In these cases, Relugolix CT had been initiated as a preoperative therapy with the objective of improving clinical status and quality of life prior to surgical intervention.

Baseline clinical and fibroid characteristics of the study population, as well as the timing of drug prescription and treatment indication (pre-surgical or indefinite), are presented in Table 1.

**Table 1:** Clinical characteristics of the study population.

Variable	**Value**
Age (years)	46.4 (5.3)
Total bleeding days	12 (7.3)
Days of heavy bleeding (SMA)	7.1(5.1)
Hemoglobine (g/dL)	11.5 (2.5)
UFS-QoL Symptom Severity Score (SSS)	27.9 (5.9)
Previous treatment	119 (83.8 %)
Number of fibroids	
*1*	66 (46.5 %)
*2*	16 (11.3 %)
*> 2*	60 (42.3 %)
Maximum fibroid diameter (cm)	5.5 (2.3)
Treatment indication	
*Long-term*	94 (66.7 %)
*Pre-surgical*	48 (33.3 %)
Timing of prescription	
*First year after commercialization*	75 (53.2 %)
*Second year after commercialization*	66 (46.8 %)

*Data are expressed as n (%) or mean±SD.

As shown in Table 2, treatment with Relugolix CT resulted in a marked and sustained improvement in clinical symptoms at both 6 and 12 months. There was a progressive reduction in symptom severity, total bleeding days, and days with heavy menstrual bleeding. Additionally, the proportion of patients achieving amenorrhea increased from approximately 52 % at 6 months to 66 % at 12 months.

**Table 2:** Clinical efficacy of Relugolix CT at 6 and 12 Months.

Variable	Baseline (n = 142)	6 Months (n = 81)	12 Months (n = 52)	p-value
Symptom Severity Score (SSS), (Mean, SD)	27.9 (5.9)	15.1(6.5)	12.8 (5.5)	< 0.05
Total bleeding days, (Mean, SD)	12 (7.3)	2.2 (4.75)	3.0 (4.75)	< 0.05
Heavy bleeding days (Mean, SD)	7.1 (5.1)	0.4 (1.2)	0.4 (1.2)	< 0.05
Amenorrhea, n (%)	0	42 (52.7 %)	33 (66 %)	< 0.05

At 6 months, patients who remained on treatment showed lower mean values for total bleeding days, heavy menstrual bleeding days, and Symptom Severity Score (SSS), compared to those who discontinued treatment. The amenorrhea rate was also higher in the continuation group (52 % vs. 24 %) (Table 3). As shown in Table 4, clinical variables at 6 months improved regardless of treatment indication (pre-surgery vs long-term) in patients who remained on treatment.

**Table 3:** Clinical variables according to treatment continuation or discontinuation at 6 months.

Variable	Continued (n = 81)	Discontinued (n = 44)	p-value
Total bleeding days, (Mean, SD)	2.2 (4.75)	13.5 (4.9)	< 0.05
Heavy bleeding days (Mean, SD)	0.4 (1.2)	7.0 (5.0)	< 0.05
SSS (Mean, SD)	15.1(6.5)	21.2 (10.3)	< 0.05

**Table 4:** Clinical variables according to treatment indication at 6 months in patients who continued the treatment.

	Pre-surgical (n = 24)	Long term (n = 57)
Total bleeding days, (Mean, SD)	14 (8.7)	10.8 (6.7)
Heavy bleeding days (Mean, SD)	8.1 (6.1)	7.0 (4.5)
SSS (Mean, SD)	26.9 (6.0)	28.7 (5,5)
Maximum fibroid diameter (cm)	5.4 (2.7)	5,5 (2.3)

*No statistically significant differences were observed (all p > 0.05)

In the multivariable linear regression analysis, treatment continuation was significantly associated with a reduction in both total bleeding days and days of HMB. None of the other predictors, including age, fibroid size, fibroid number, or fibroid type, showed statistically significant associations with either bleeding outcome (Table 5).

**Table 5:** Regression Models for Total and Heavy Menstrual Bleeding Days at 6 months.

Predictor	Total Bleeding Days – Estimate (95 % CI)	p-value	Heavy Bleeding Days – Estimate (95 % CI)	p-value
Treatment continuation (Yes vs. No)	-4.29 (-7.70−0.89)	0.01	-2.76 (-4.41 −1.11)	0.01
Age	-0.10 (-0.33–0.13)	0.38	0.04 (-0.07–0.15)	0.46
Max fibroid diameter (mm)	-0.01 (-0.06–0.03)	0.59	-0.02 (-0.04–0.00)	0.12
2 fibroids vs. 1	2.79 (-1.64–7.23)	0.21	-0.42 (-2.70–1.84)	0.70
≥ 3 fibroids vs. 1	2.14 (-0.94–5.24)	0.17	-0.34 (-1.85–1.17)	0.65
Submucosal vs. Intramural	-0.72 (-3.88–2.42)	0.64	-0.06 (-1.57–1.43)	0.92
Subserosal vs. Intramural	-1.16 (-6.10–3.76)	0.63	1.32 (-1.02–3.67)	0.26
Intercept	10.92 (-1.68–23.53)	0.08	2.51 (-3.51–8.55)	0.40

After 6 months, 61 patients had discontinued the prescribed treatment, with planned surgery being the most common reason (Table 6).

**Table 6:** Reasons for Treatment Discontinuation at 6 Months.

Reason	N (%)
Planned surgery	17 (27,9 %)
Patient-perceived lack of efficacy	15 (24,6 %)
Poor tolerability	13 (21,3 %)
Unknown reason	12 (19.7 %)
Patient decision ("did not want")	3 (4.9 %)
Menopause	1 (1,6 %)

Among patients who discontinued treatment by the 6-month follow-up, a subgroup analysis was conducted excluding those with a previously scheduled surgical procedure, for whom treatment had been completed as planned. Within the remaining group, the most frequent reason for discontinuation was patient-perceived lack of efficacy, followed by poor tolerability.

Of the 142 patients who were prescribed the treatment, only 15 (10.5 %) discontinued due to patient-perceived lack of efficacy.

**Fig. 1:**
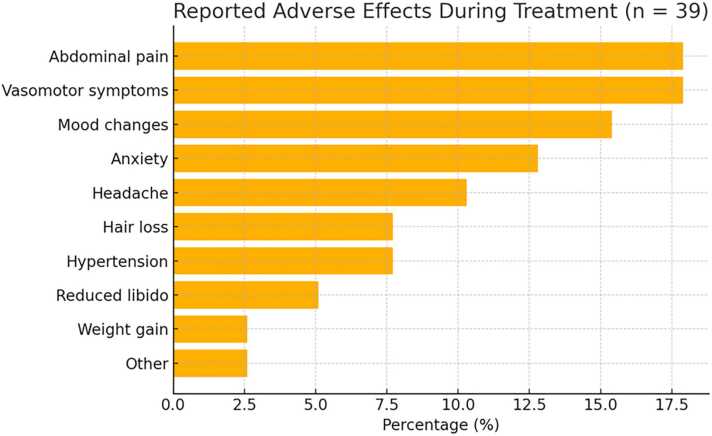
Reported adverse effects during treatment.

A total of 39 patients reported adverse events. The most commonly reported were abdominal pain and vasomotor symptoms, followed by mood changes, anxiety, and headache (Fig. 1).

In the 52 patients who completed one year of treatment with Relugolix CT [mean age 46.6 (5.4)] bone densitometry of the lumbar spine and hip was performed. Osteopenia was identified in 10 % of patients at the lumbar spine and in 18 % at the hip. No cases of osteoporosis were observed at either site. The mean T-score was + 0.3 (range: −2 to +2) in the lumbar spine and −0.2 (range: −1.7 to +5) in the hip (Table 7).

**Table 7:** Bone densitometry results after a year of treatment.

	Cases. N = 52	%
**Lumbar spine**		
Osteopenia (T score = −1–2,5)	5	10
Osteoporosis (T Score = <-2,5)	0	0
**Hip**		
Osteopenia (T score = −1–2,5)	9	18
Osteoporosis (T Score = <-2,5)	0	0

Among the 81 patients with an active prescription for Relugolix CT at the 6-month evaluation, treatment continuation was higher in those with a pre-surgical indication (91.7 %) than in those prescribed the medication for long-term use (76.4 %). However, this difference was not statistically significant, and adherence remained relatively high overall Similarly, patients who initiated treatment in the second year after commercialization had a higher continuation rate (92.9 %) than those who started in the first year (74.5 %). Patients who had discontinued treatment before completing 6 months, or for whom Relugolix CT was no longer prescribed at that time, were excluded from this analysis (Table 8).

**Table 8:** Treatment Continuation According to Indication and Year of Prescription.

Group	Did not continue	Continued	p
Indication			
*Long-term (indefinite)*	13 (23.6 %)	42 (76.4 %)	
*Pre-surgical*	2 (8.3 %)	22 (91.7 %)	0.1
Year of prescription			
*2023 (Year 1)*	13 (25.5 %)	38 (74.5 %)	
*2024 (Year 2)*	2 (7.1 %)	26 (92.9 %)	< 0.05

## 4 Discussion

The use of Relugolix CT in our real-world gynecological population has proven effective in managing symptoms associated with uterine fibroids. Significant clinical improvements were observed in all bleeding-related variables, and in patient-reported outcomes using the UFS-QoL SSS. Treatment was generally well tolerated, with adverse effects mostly mild and infrequent.

At both 6 and 12 months of treatment, whether prescribed pre-surgically or for indefinite use, Relugolix CT significantly reduced total menstrual bleeding days and days with HMB. Nearly half of the patients achieved amenorrhea. These results are consistent with the LIBERTY 1 and 2 trials, which —despite using different primary endpoints—reported similar improvements in menstrual bleeding, with amenorrhea rates of 50–52 % at 6 months and 71 % at 12 months. In our cohort, amenorrhea was achieved in 52 % and 66 % of patients at the same time points, respectively. Furthermore, symptom scores and quality-of-life also improved progressively from 6 to 12 months, in line with LIBERTY findings.

At the 6-month follow-up, 31 women with a pre-surgical indication had not yet undergone surgery. Given that improvements in bleeding outcomes were observed regardless of the initial treatment indication, these findings raise the question of whether some patients initially considered for surgery might reconsider or even avoid surgical intervention after experiencing clinical benefit with medical therapy.

Unlike the LIBERTY studies, which reported significant reductions in fibroid and uterine volume, our analysis lacks data on volumetric changes, as fibroid size was not part of routine follow-up. This reflects our pragmatic approach, where Relugolix CT is primarily prescribed for symptom control, while volume reduction is usually pursued through surgical or ablative interventions like radiofrequency ablation.

Tolerability in our population was similar to previous phase III trials, with 34.5 % of patients reporting adverse events—comparable to 30.9 % in LIBERTY 1 and 41.4 % in LIBERTY 2. However, the type of adverse effects varied. In our population, abdominal discomfort and hypoestrogenic symptoms (e.g., hot flushes, irritability, mood lability) were most frequent, while LIBERTY trials reported more headache and hypertension. These differences may stem from varied methods of adverse events collection—systematic via e-diaries and structured follow-up in trials versus open-ended interviews during routine follow-up in our setting—and may also reflect cultural or social influences on symptom reporting.

One of the most important safety concerns in long-term GnRH antagonist therapy is its potential impact on BMD. In the LIBERTY extension study, no clinically significant BMD reductions were reported after 12 or 24 months, though a mean reduction of 0.8 % and > 3 % loss in only 21 % of patients were noted. In our study, baseline BMD was not routinely performed, as it is only recommended in the presence of risk factors of osteoporosis. None of our premenopausal patients had such risk factors. However, densitometry was mandatory at 12 months for treatment renewal under the public health system. Among 52 patients who completed one year of treatment, no osteoporosis was identified. Osteopenia occurred in 10 % of patients at the lumbar spine and in 18 % at the hip—figures that are consistent with osteopenia rates in the general perimenopausal female population within this age range 9, 10.While baseline BMD values would have helped quantify changes, our findings are consistent with published literature and reinforce the skeletal safety profile of Relugolix CT.

A total of 61 patients discontinued treatment in our study, mainly due to elective surgery and patient-perceived lack of efficacy. LIBERTY trials did not report overall dropout rates but noted that only 3.9 % discontinued due to adverse effects. In this regard, it is noteworthy that only 10.5 % of the patients to whom the drug was prescribed discontinued it due to a patient-perceived lack of efficacy.

In our study, the initial treatment indication—pre-surgical vs. indefinite—emerged as an important factor influencing treatment continuation. Notably, no meaningful baseline clinical or fibroid-related differences were found between groups, yet discontinuation was nearly three times higher among indefinite users. However, this trend did not reach statistical significance—likely due to the small number of pre-surgical patients still awaiting surgery at 6 months.

Treatment continuation varied according to the year of prescription, suggesting that, prescription timing impacted adherence. Patients who initiated treatment in 2024 had significantly lower dropout rates than those who started in 2023. This likely reflects a clinician learning curve, as familiarity with the medication increased, so did accuracy in patient selection and communication about treatment expectations and potential side effects. This underscores the importance of individualized patient counseling. Understanding each patient’s preferences, expectations, and treatment goals is vital for improving adherence and clinical outcomes.

A major strengths of this study is its reflection of everyday clinical reality. Patients were managed under standard care conditions, without the restrictive inclusion criteria or close monitoring typical of clinical trials. This enhances the generalizability of our findings. However, limitations include a relatively small sample size, significant loss to follow-up, and the lack of systematic data collection at 12 months. Therefore, while results are promising, they should be interpreted cautiously and confirmed in larger, prospective studies with longer follow-up periods.

## 5 Conclusion

Relugolix CT is an effective and well-tolerated treatment for symptomatic uterine fibroids in real-world settings, offering significant symptom relief and a favourable safety profile, Relugolix CT reflects long-term skeletal safety consistent with previous clinical trial data. These findings underscore the importance of personalized counselling and clinician experience in improving treatment adherence to guarantee an adequate management of these patients.

## CRediT authorship contribution statement

**A. Santalla-Hernández:** Supervision, Project administration, Formal analysis. **M. Naveiro-Fuentes:** Formal analysis, Data curation, Conceptualization. **N. Esquinas Orellana:** Investigation, Data curation. **LM Benítez Cejas:** Methodology, Investigation, Data curation. **M. García Rivera:** Writing – original draft, Formal analysis. **J. Fernández-Parra:** Writing – original draft, Supervision, Investigation.

## Declaration of Competing Interest

The authors declare that they have no known competing financial interests or personal relationships that could have appeared to influence the work reported in this article.
